# MiR-223-3p Alleviates Vascular Endothelial Injury by Targeting IL6ST in Kawasaki Disease

**DOI:** 10.3389/fped.2019.00288

**Published:** 2019-07-24

**Authors:** Xiang Wang, Yue yue Ding, Ye Chen, Qiu qin Xu, Guang hui Qian, Wei guo Qian, Lei Cao, Wan ping Zhou, Miao Hou, Hai tao Lv

**Affiliations:** ^1^Department of Cardiology, Children's Hospital of Soochow University, Suzhou, China; ^2^Department of Pediatrics, The Affiliated Huaian No.1 People's Hospital of Nanjing Medical University, Huaian, China; ^3^Pediatric Research Institute of Soochow University, Suzhou, China

**Keywords:** Kawasaki disease, MicroRNA-223-3p, IL6ST, vascular endothelial damage, STAT3

## Abstract

**Background:** Kawasaki disease (KD) is a self-limiting illness with acute systematic vascular inflammation. It causes pathological changes in mostly medium and small-sized arteries, especially the arteria coronaria, which adds the risk of developing coronary heart disease in adults.

**Materials and methods:** We detected the miR-223-3p expression in 30 KD patients combined with 12 normal controls using miRNA microarrays and RT-PCR. A KD mouse model was constructed using Candida albicans water insoluble substance (CAWS). We also checked the miR-223-3p's expression using qRT-PCR. The Luciferase reporting system was implemented to validate the correlation between miR-223-3p and Interleukin-6 receptor subunit beta (IL-6ST). TNF-α was used to stimulate human coronary artery endothelial cells (HCAECs), and miR-223-3p activator or inhibitor and KD serum were used to treat HCAECs. A Western blotting automatic quantitative analysis protein imprinting system was used to test the expression of signal transducer and the activator of transcription 3 (STAT3), phosphorylated-signal transducer and the activator of transcription 3 (pSTAT3) and NF-κB p65.

**Results:** Clinical trials found that miR-223-3p expressions were markedly different (more than 2-fold) between the acute KD group and the control group. E-selectin and intercellular cell adhesion molecule-1 (ICAM-1) levels were also significantly higher (about 2-fold) in KD especially with coronary artery lesions. MiR-223-3p could alleviate vascular endothelial damage in KD mice, and IL-6 (Interleukin-6), E-selectin and ICAM-1 were simultaneously negative. The values of IL-6, E-selectin, and ICAM-1 mRNA expressions decreased, while the value of IL-6ST was increased between the agonist treated mice and KD mice. The RT-qPCR consequences displayed that miR-223-3p explored the highest expression on the third day in both the KD mice as well as the agonist group. MiR-223-3p can directly combine with IL-6ST 3' untranslatable regions (UTR) and held back the IL-6's expression. Overexpression of miR-223 down regulated IL6ST expression and decreased the expression of p-STAT3 and NF-κB p65, while the miR-223 inhibitor could reverse the above process.

**Conclusion:** MiR-223-3p is an important regulatory factor of vascular endothelial damage in KD and could possibly become a potential target of KD treatment in the future.

## Introduction

Kawasaki disease (KD) is an acute systemic vascular inflammatory pathema, which largely impacts medium and small-sized arteries, particularly the arteria coronaria (1). Without early intervention, about 20% of children with KD may develop coronary artery aneurysms (2). Studies have found that the deregulation of innate immunity, vascular endothelial growth factor (VEGF) A and other reasons such as infectious factors are closely related to the pathogenesis of KD (3–5). However, the etiology of KD remains unclear. This underlines the need to reveal the potential pathogenesis mechanisms of KD, thus providing novel therapeutic targets for the condition.

MicroRNAs (miRNAs) are 18–25 small nucleotide non-coding RNAs which can adjust and control the genetic expression by imperfect hybridization to the 3′ untranslatable regions (UTR) of target mRNAs (6). miRNAs regulate nearly one third of the human gene function, and are involved in cell multiplication, growth, apoptosis, and the metabolism of important biological processes (7). For instance, in acute KD, microRNA-93 has been declared to regulate the expression of VEGF and is possibly involved in the pathogenesis of arteritis (8).

MiR-223 is deemed to be a cell–specific, hematopoietic lineage miRNA, which are exclusively expressed in blood cells such as blood platelets and leukocytes which are derived from bone marrow (9). Recent research has revealed that miR-223 participates in the development of atherosclerosis in experimental mice suffering from chronic kidney disease (CKD) (10). It has also been noted that as a receptor of the insulin-like growth factor 1, miR-223 can promote human umbilical vein endothelial cells apoptosis (11). These findings reveal that miR-223 could also be involved in vascular diseases such as KD which is explored in the present study. Results show that miR-223-3p plays a protective role against endothelial injury in KD, by targeting IL6ST and by regulating the STAT3-NF-κB signaling pathway, making it a potential target for the diagnosis and treatment of KD.

## Materials and Methods

### Patient Characteristics

Plasma specimens were collected from KD children (*n* = 6; two males and four females, mean age, 24 months) and healthy controls (*n* = 6; two males and four females; mean age, 18 months) for miRNA microarray hybridization. Plasma specimens, used for the investigation, were collected from 30 patients [18 males and 12 females; median age: 18 months (range: 4–90 months)] with acute and subacute KD. Considering fever onset as the first day of the KD course, acute stage (prior treatment of intravenous human immunoglobulin, range: 1–10 days), subacute stage (post-treatment of intravenous human immunoglobulin, normothermia, range: 11–21 days). These groups were subdivided into coronary artery lesions (CALs) (*n* = 10) and no coronary artery lesions (nCALs) (*n* = 20) subgroups. CALs mainly include occlusion, localized stenosis, segmental stenosis, dilatation, and aneurysms during coronary angiography. In our study, echocardiography was utilized to detect cardiac vasodilation and coronary aneurysms at admission. The maximum inner diameters of both the left and right coronary arteries were measured in the two groups. CALs were defined as those whose maximum internal diameters were larger than 3.5 mm in those aged >9 y, larger than 3.0 mm in those aged 3–9 y, and larger than 2.5 mm in those aged <3 y. The diagnosis of KD was made on the basis of the criteria developed in 2004 by the American Heart Association. All patients, including those with KD and febrile controls, from the Cardiology Department, of the Children's Hospital of Soochow University (Suzhou, Jiangsu Province, China) were enrolled, from August 2015 to August 2016. In addition, plasma samples were collected from 24 controls comprising 12 healthy controls after routine physical examinations (male: *n* = 8; female: *n* = 4; median age:30 months) (12–84 months) and 12 febrile patients with proven respiratory infection (male: *n* = 7; female: *n* = 5; median age: 29 months) (range: 8–81 months). The whole blood sample (2 ml) was drawn into an EDTA-containing tube. After being centrifuged for 15 min at 3,500 rpm to spin down the blood cells, the supernatant was shifted into a clean microcentrifuge tube, followed with the second centrifugation to absolutely eliminate the cellular components. Plasma was then aliquoted and stored in a refrigerator at −80°C partitions for further use. All the study participants were obliged to provide signed informed consent, and the research was undertaken in light of the institutional ethics committees approved protocol and HIPAA regulations.

### CAWS KD Mice Model

Kawasaki disease was simulated by intraperitoneal injection (20 mg/Kg; 5 consecutive days) of Candida albicans water-soluble fraction (CAWS) to C57BL/6 male mice of 4–6 weeks of age. miR-223-3p activator was purchased from GeneCopoeia (Rockville, MD, USA), and dispersed in PBS. KD mice received a tail vein injection of miR-223-3p activator (1 mg/kg). Seventy-two mice were divided into three groups randomly: ① The control group: normal saline intraperitoneal injection (0.1 ml;5 consecutive days). ② The KD group: CAWS intraperitoneal injection (0.1 ml, 20 mg/Kg; 5 consecutive days). ③ MiR-223-3p activator group: CAWS intraperitoneal injection (0.1 ml, 20 mg/Kg; 5 consecutive days) and tail vein injections of miR-223-3p activator (1 mg/kg, in the fifth day). Each group was sacrificed on the first day (6 day), third day (8 day), fifth day (10 day), seventh day (12 day), and tenth day (15 day), respectively. Post-sacrifice, whole blood from mice was drawn into EDTA-containing tubes and serum was extracted following the same protocol as with a patients' blood. Observation of the aorta was achieved under HE staining and a scanning electron microscope (SEM).

### Cell Culture and Treatment

HCAECs were purchased from American Type Culture Collection (ATCC) (Manassas, VA, USA) and were cultured to confluence in RPMI 1640 medium (Gibco; Thermo Fisher Scientific, MA, USA) mixed with both 1% penicillin/streptomycin and 10% fetal bovine serum, in a humidified atmosphere with 5% CO_2_ and the temperature was maintained at 37°C. HCAECs were then randomly and transiently transfected with miR-223-3p activators, negative control oligonucleotides or miR-223-3p inhibitors (all were compounded by Shanghai Genechem Co. Ltd, Shanghai) acting on Lipofectamine 2000 reagent (Invitrogen; Thermo Fisher Scientific, Inc). HCAECs were seeded in a six-well plate, at 1 × 10^5^ cells/ml. Transfection was performed after the cells reached 70% confluence. 20 nmol/L of miR-223 activators, negative control or miR-223 inhibitors were dispersed in Opti-MEM (Thermo Fisher Scientific) with twice the volume of Lipofectamine 2000. After 15 min quiescence at room temperature, the mixture was added into HCAECs 6 h post transfection, cells were given a PBS wash and the medium was replaced. The cells were then incubated in the aforementioned conditions and harvested after 24 h. For the KD serum stimulation assay, HCAECs were stimulated with acute phase KD serum and the healthy control group, respectively. Prior to use, we first used a 30 min long bath in 56°C water to inactivate the serum from holding back any immune response. Then the HCAECs were incubated in the serum-containing media for 48 h, after which they were harvested, and total mRNA was extracted from them. Finally, the RT-qPCR was utilized to celebrate the miR-223-3p expression level.

### Reverse Transcription and Real-Time PCR

An MiRNeasy Serum/Plasma Kit (Qiagen) and miRNeasy Mini Kit (Qiagen, Hilden, Alemania) were used to extract the total RNA from the serum samples and the cells, respectively. An MiRNA specific Taqman MicroAssay (#4427975; ID 002619; Applied Biosystems) and Taqman miRNA Reverse Transcription Kit (Applied Biosystems) were used for reverse transcription. Specific primers used for all the microRNAs were obtained from Applied Biosystems [hsa-miR-16 (internal reference): upstream primer sequence 5′TAGCAGCACGTAAATATTGGCG3′; hsa-miR-223-3p: upstream primer sequence 5′TGTCAGTTTGTCAAATACCCCA3′;hsa-miR-765:upstream primer sequence 5′TGGAGGAGAAGGAAGGTGATG3′;hsa-miR-33b-3p:upstream primer sequence 5′CAGTGCCTCGG CAGTGCAGCCC3′; The downstream primer comes with the Qiagen kit]. These reactions were all carried out in duplicate in the 96-well plates, and the data were evaluated with the help of 7900HT Fast Real-Time PCR systems (Applied Biosystems).

### MTT Assay

The proliferation rate of the transfected HCAECs was detected by the MTT assay. Briefly, 1 × 10^4^ cells/well was suspended in 200 μl culture medium and seeded in a plate with 96-wells. After 24 h of cell seeding, we first used the serum-free medium containing 5 μg/ml MTT to replace the culture medium. Then, after further incubation for 4 h, the serum-free medium was replaced by DMSO, mixed well, and finally the SpectraMax 190 (Molecular Devices in Sunnyvale, CA, USA) was used to measure the optical density by recording the absorbance at 490 nm.

### Luciferase Assay

HCAECs were seeded in a 24-well plate first. miR-223-3p and Mut 3′UTR or WT 3′UTR of IL6ST or the control mimics were co-transfected at the same time. After 48 h of incubation, cells were reaped using the DualLuciferase® Reporter Assay System (Promega, Madison, WI, USA) for Renilla and firefly luciferase activity assays.

### Western Blotting

RIPA lysis buffer, supplemented with complete protease inhibitor cocktail (Beyotime, China) were employed to lyse the transfected HCAECs. Then we used the BCA Protein Assay Kit (Beyotime, China) to isolate the total protein. Immediately, 30 μg of total protein in the loading buffer were loaded per lane and separated by the Sodium Dodecyl Sulfate Polyacrylamide Gel Electrophoresis (SDS-PAGE) for immunoblotting. Polyvinylidene difluoride (PVDF) membranes were then used to transfer those separated proteins. After blocking the membrane for 60 min using 5% skimmed milk, we used those primary antibodies STAT3 (ab119352, abcam), pSTAT3 (phospho Y705, ab76315, abcam), NF-kB p65 (ab16502, abcam), β-actin (66009-1-Ig, Proteintech), IL6ST (ab202850, abcam) to incubate with the membranes overnight at 4°C. On the next day, post-wash, the membranes were incubated at room temperature with secondary antibodies for 2 h, then we used chemiluminescence to detect the protein bands.

### Statistical Analysis

Data was presented as the mean ± standard deviation ($\bar{x}\pm$SD) or median (range: minimum, maximum) from at least three independent experiments. One-way analysis of variance (one-way ANOVA) with the Newman-Keuls comparison test was employed to determine the significant difference between groups. Statistical analysis was carried out using SPSS 17.0 software (IBM Corporation, Armonk, NY, USA). A level of *p* < 0.05 was considered as statistically significant.

## Results

### The Expression Level of MiR-223-3p Is Upregulated in the Serum of Patients With KD

**Figure 1:**
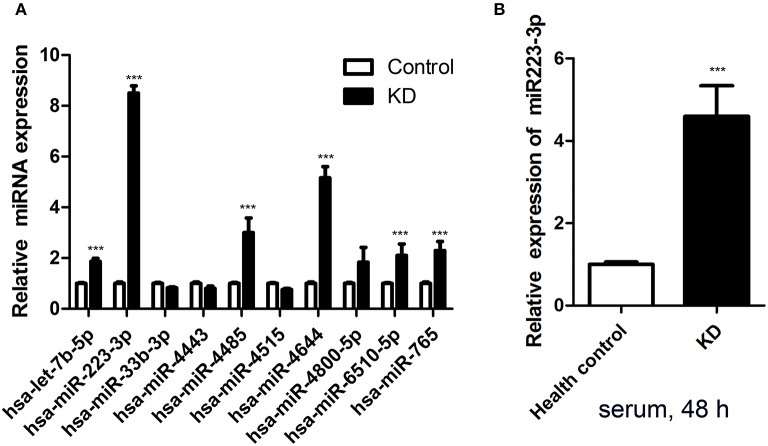
Serum miR-223-3p was increased in patients with KD. **(A)** The result of Affymetrix microRNA microarrays for the expression of 10 miRNAs in Kawasaki disease acute stage plasma. **(B)** Expression of miR-223-3p was measured in HCAECs by RT-qPCR at 48 h following culture with 2 ml serum from KD patients and healthy controls (*n* = 3). Data are presented as mean ± standard error of mean, ***P < 0.001.

MiRNA microarrays revealed seven prominently upregulated miRNAs (hsa-let-7b-5p, hsa-miR-223-3p, hsa-miR-765, hsa-miR-4485, hsa-miR-4644, hsa-miR-4800-5p, sa-miR-6510-5p) and three remarkably downregulated miRNAs (hsa-miR-33b-3p, hsa-miR-4443, and hsa-miR-4515) among the plasma samples of six acute KD patients, compared with the levels detected in the healthy groups. So, the expression levels of these 10 miRNAs were accessed subsequently. Amongst 10 different miRNAs assessed, miR-223-3p was proved to be dramatically increased in the KD serum, almost about 9-fold higher (*p* < 0.001) in contrast to the healthy control group (Figure 1A).

### HCAECs Stimulated With KD Serum

As an *in vitro* model, HCAECs were applied to study the vascular endothelium's function in our research. In order to test whether KD serum could increase miR-223-3p, KD acute stage serum was employed as a stimulant. The miR-223-3p expression level was determined followed by 48 h stimulation. The results revealed that there was a striking increase of the miR-223-3p level in those cultured with KD serum cells, but not for those in the healthy control group (Figure 1B). A probable mechanism of miR-223-3p was thus indicated in affecting the endothelial cells during the pathogenesis of KD.

### Increased Expression of miR-223-3p Was Peculiar in Acute KD

**Figure 2:**
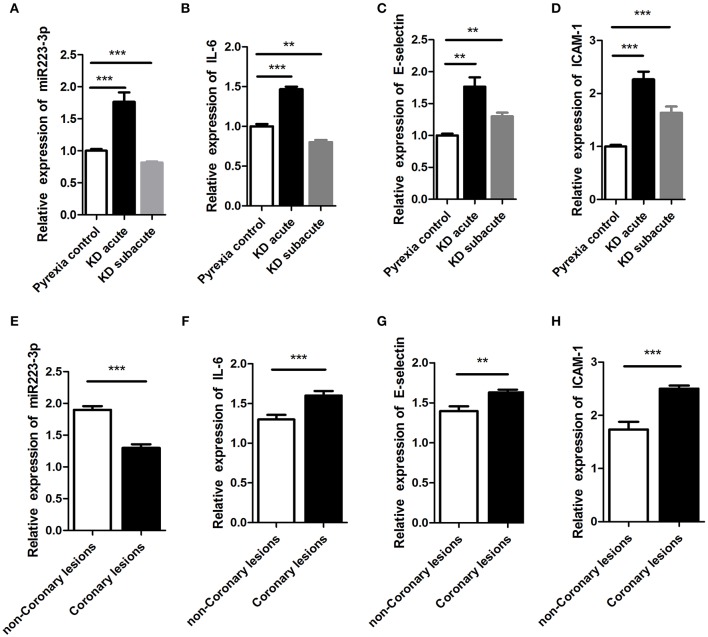
The expression of miR-223-3p was seen to increase in acute stage of KD. panel **(A–D)** represent the relative expression levels of **(A)** miR-223-3p, **(B)** IL-6, **(C)** E-selectin, and **(D)** ICAM-1, respectively, in Pyrexia control, acute KD, and subacute KD. This figure represents the relative expression of **(E)** miR-223-3p, **(F)** IL-6, **(G)** E-selectin, and **(H)** ICAM-1 in coronary lesions group and non-coronary lesions group of KD. **P < 0.01 and ***P < 0.001.

We assessed the miR-223-3p expression level during both the acute and subacute stages of KD. Results displayed that the miR-223-3p expression level augmented 2-folds in the acute stage of KD (*p* < 0.001) and declined to 70% in the subacute stage of KD (*p* = 0.021), in comparison with the pyrexia control group (Figure 2A). Further, in acute KD serum, the miR-223-3p expression level was lower in the serum sample obtained from the CAL group than that in the nCAL group (Figure 2E). Similarly, we measured the variation in the expression levels of IL-6, ICAM-1, and E-selectin during different stages of KD at the same time. The trend was similar to miR-223-3p expression, where all the three molecules were found to be increasingly expressed in the acute stage KD serum sample while decreased in the subacute stage (Figures 2B–D). However, in contrast to the miR-223-3p expression levels in the acute KD serum, the expression of the three molecules increased in the CAL serum compared to that in the nCAL serum (Figures 2F–H).

### IL6ST Was Found to be a Target Gene of miR-223-3p

**Figure 3:**
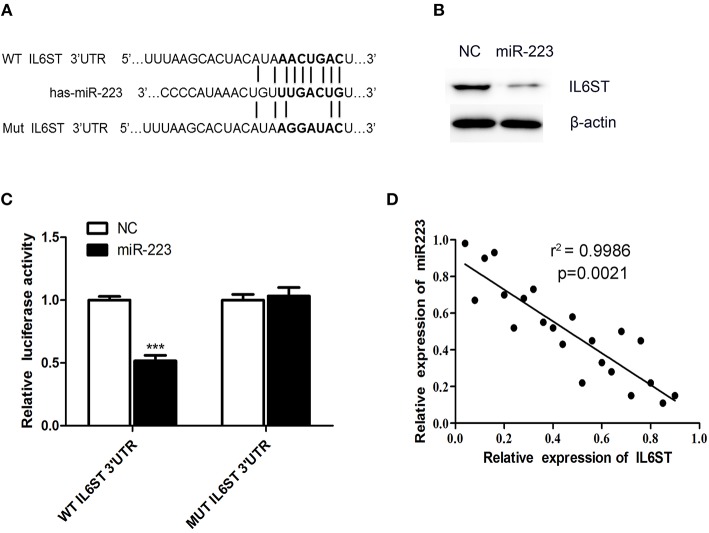
IL6ST is a target gene of miR-223-3p. **(A)** Shows the IL6ST 3′UTR-containing reporter construct. Schematic representation of the miR-223-3p sequence, putative miR-223-3p targeting site in the 3′UTR of IL6ST, and the generated mutant IL6ST 3′UTR. **(B)** The western blot analysis shows that the cells transfected with miR-223 showed lower expression of IL6ST protein. **(C)** Luciferase reporter assay indicates the inhibitory effect that miR-223 has on the luciferase activity of IL6ST-3′UTR. **(D)** Correlation of the expression of IL6ST to that of miR-223-3p in KD samples using simple linear regression analysis. ***P < 0.001.

To elucidate the underlying mechanism of miR-223-3p, we have planned to explore the miR-223-3p target genes with the help of the TargetScan bioinformatics algorithm. Based on putative target sequences at position 182-205 of the IL6ST 3′UTR, our analysis uncovered the fact that IL6ST is a potential target of miR-223-3p (Figure 3A). We then engineered luciferase reporter constructs containing the mutant (Mut) 3′UTR and the wild-type (WT) 3′UTR of the IL6ST to ascertain whether IL6ST can serve as a direct target spot of the miR-223-3p. The luciferase reporter assay data proves that miR-223-3p can remarkably decline the luciferase activity of the IL6ST 3′UTR but not the mutant IL6ST 3′UTR (Figure 3C). Additionally, the western blot analyses also revealed the fact that miR-223-3p overexpression dramatically downregulated the expression of IL6ST in HCAECs (Figure 3B). The relationship between IL6ST and miR-223-3p was further analyzed by detecting the relative expression level of IL6ST, and a significant negative correlation was observed between the miR-223-3p expression and IL6ST using the Spearman's correlation analysis (Figure 3D). Above all, we can conclude that IL6ST is the target gene of miR-223-3p in KD.

### miR-223-3p Regulated the Inflammatory Factor Level in KD Mice

**Figure 4:**
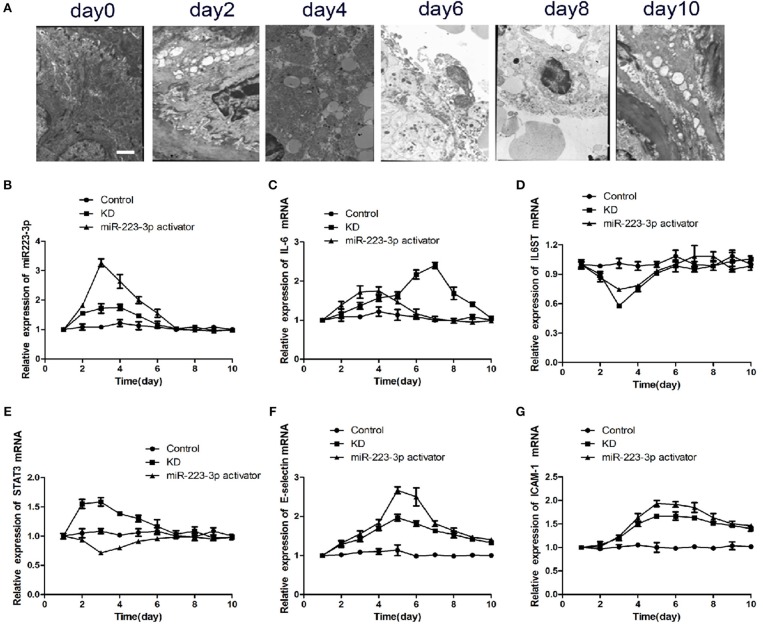
miR-223-3p regulated the inflammatory factor levels in KD mice. **(A)** Representative SEM images of CAWS induced KD mice model at different stages. The Scale bar was “×5000.” Relative expression levels of **(B)** miR-223-3p, **(C)** IL-6, **(D)** IL-6ST, **(E)** STAT3, **(F)** E-selectin, and **(G)** ICAM-1 in control mice, KD mice, and miR223-3p activator treated KD mice with respect to time.

Six of the 24 rats died in the CAWS intraperitoneal injection group, and the mortality rate was between 10 and 30%. The majority of mice were found to have intestinal adhesion, intestinal mucosal swelling, obstruction, and even necrosis (Supplementary Figure 1A). The hair color of the CAWS groups became yellow and disorderly and they experienced a loss in appetite (Supplementary Figures 1B,C). One third of the intraperitoneal injection sites had visible scabs, and local skin erosion was visible in about 1/4 of sites (Supplementary Figures 1D,E). We observed two cases of aneurysmal dilatation on the seventh day in the KD group (Supplementary Figures 1F,G). By the tenth day, the mental reaction of mice had improved in both groups, the food intake increased, and their hair color became dark and bright. One case of membranous peeling was observed at the end of the toe in the KD group (Supplementary Figure 1H). While two of the 24 rats died in the CAWS intraperitoneal injection combined with the miR-223-3p activator tail vein injection group. Obviously the mortality rate in the control group was markedly lower than that of the CAWS group. The results of these pathological specimens suggested that inflammation, thrombosis, and a thickening of the vascular wall were observed in the heart tissue of mice in the KD group, which is consistent with the pathological changes observed in Kawasaki disease vasculitis (Supplementary Figures 2A–H). However, there was no more severe vasculitis caused by lactobacillus caseate cell wall extract (LCWS) and the pathological picture was not so typical. In addition, we also compared the pathological discrepancy with HE staining at different time nodes between the KD and the miR-223-3p activator group, but the differences were not so obvious. As is evident by the SEM images (Figure 4A), after a period of 4 days post induction, the aorta showed intimal inflammation and the simultaneous appearance of a cavitation endothelial injury. After 6–8 days post induction, the vascular endothelium is shed off, cytoplasmic vacuolation occurs, and the endothelial injury reaches its peak. After a period of 10 days post induction, the inflammatory lesions begin to recede, and the mice enter the recovery phase.

To verify the particular function of miR-223-3p in vascular endothelial injury *in vivo*, the KD mice were injected with miR-223-3p activator. The RT-qPCR results revealed that miR-223-3p explored the highest expression on the third day in both the KD mice as well as the miR-223-3p activator treated KD mice. Both showed a subsequent decrease to the control level on the seventh day (Figure 4B). Although IL-6 showed a maximum expression on the third day, which then decreased to the control level on the seventh day. The highest levels of IL-6 were noted on the seventh day, after intraperitoneal injection with the miR-223-3p activator (Figure 4C). IL-6ST showed minimal expression on the third day in both the KD mice and the miR-223-3p activator treated KD mice, and then showed an increased expression compared to the control level on the seventh day (Figure 4D). Our data revealed that the expression of STAT3 mRNA was highest on the third day in KD mice while the value was the lowest in mice who received a miR-223-3p activator injection (Figure 4E). The expression levels of E-selectin and ICAM-1 were also detected, and the results indicated that E-selectin and ICAM-1 both showed the highest expression level on the fifth day and their expression decreased subsequently. However, a notable feature was that the level of E-selectin and ICAM-1 would not decrease to the control level even 10 days post treatment (Figures 4F,G).

### miR-223 Inhibited the Expression of IL-6ST in TNF-α

**Figure 5:**
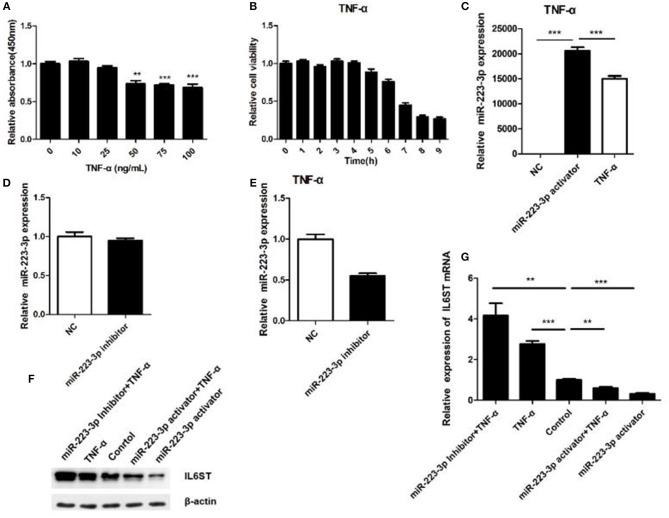
Relationship between miR-223-3p and IL-6ST in TNF-α treatment. **(A)** Effect of different concentrations of TNF-α on the viability of HCAECs detected by the MTT assay. **(B)** The effect of TNF-α (concentration) on cell viability calculated for HCAECs with different time periods of exposure. The relative expression level of miR-223-3p post treatment with miR-223-3p and miR-223-3p activator combined with TNF-α. **(D)** miR-223-3p inhibitor. **(E)** miR-223-3p inhibitor and TNF-α. **(F)** The western blot indicates that the level of expression IL-6ST was least when treated with miR-223-3p and maximum when treated with the inhibitor in the presence of TNF-α. **(G)** Relative expression analysis of IL-6ST mRNA expression. *P < 0.05, **P < 0.01, and ***P < 0.001.

TNF-α being one of the most important inflammatory factors, was used for inducing inflammatory injury. We thus investigated the biological function of miR-223-3p in TNF-α treated HCAECs. First we disposed the HCAECs with different concentrations of TNF-α for 12 h, then the cell viability was detected, respectively. The result showed that 50 ng/ml TNF-α could significantly decrease the viability of HCAECs concentrations while when concentrations of TNF-α were higher than 50 ng/ml it could not decrease the cell viability any further (Figure 5A). A time-based experiment showed that cell viability began to decrease after being treated by 50 ng/ml of TNF-α for a period of 6 h. And when treated for 8 h or longer, it showed no further decrease in the cell viability anymore (Figure 5B). Thus, HCAECs were designed to be treated with 50 ng/ml TNF-α for 8 h in further experiments. Treatment of HCAECs with miR-223-3p activator showed that TNF-α could partially inhibit miR-223-3p expression under the influence of the miR-223-3p activator (Figure 5C). While the decrease of the miR-223-3p mRNA expression level was not significant (*p* > 0.05) in contrast to the control group (Figure 5D), this may possibly be due to the fact that the basic expression level of miR-223-3p was too low. However, when treated with TNF-α 50 ng/ml, the miR-223-3p inhibitor caused a dramatical decrease in the expression level of miR-223-3p (Figure 5E). The expression level of IL6ST mRNA was also detected and the results are as indicated in Figures 5F,G. The miR-223-3p activator significantly decreases the expression of IL6ST, when co-treated with TNF-α. IL6ST expression is much lower in this case compared to when treated with the miR-223-3p activator alone. The miR-223-3p inhibitor was seen to partially increase the expression of IL6ST. However, when co-treated with TNF-α, a slight decrease in the expression of IL-6ST was observed. The above results suggest that miR-223-3p significantly down-regulates the expression of IL-6ST in inflammatory conditions induced by TNF-α.

### miR-223-3p Regulated STAT3 and NF-κB p65 Signaling Pathway

**Figure 6:**
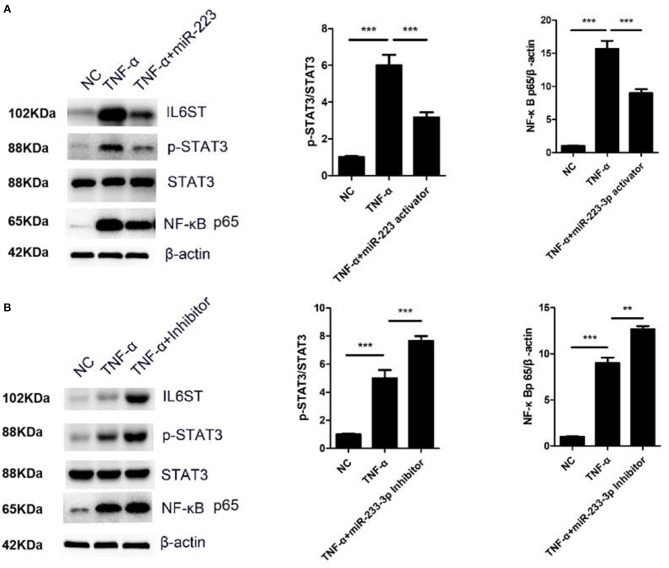
miR-223-3p regulated STAT3 and NF-κB signaling pathway. **(A)** Western blot detection of the expression of p-STAT3/STAT3 and NF-κB p65 after miR-223-3p overexpression. **(B)** Western blot detection of the expression of p-STAT3/STAT3 and NF-κB p65 after treatment with miR-223-3p inhibitor. **P < 0.01 and ***P < 0.001.

IL-1 and TNF-α can stimulate the activity of NF-κB to promote the secretion of IL-6 (12). The IL-6/IL6ST-STAT3 signaling pathway has been reported to be very crucial for inflammatory regulation (12). In the present study, the HCAECs were first treated with TNF-α, and the IL6ST expression was then suppressed by miR-223-3p. As expected, compared with the TNF-α induced group, miR-223-3p could significantly decrease the expression level of pSTAT3 and NF-κB p65 (Figure 6A). On the contrary, miR-223-3p inhibition could observably enhance the expression of pSTAT3 and NF-κB p65 at the same time (Figure 6B). In conclusion, these results imply that miR-223 can hold the activation of the STAT3 signaling pathway back, by targeting the inhibition of IL6ST, thus inducing vascular endothelial cell injury.

## Discussion

In recent years, KD has gradually turned into one of the major reasons for acquired heart diseases that affect children, which has attracted increased attention, while the vascular injury complication associated with KD has made it an important risk factor for adult ischemic heart disease (13). Unfortunately, our present understanding about the exact pathogenesis of vascular damage in KD is very limited and insufficient, and effective therapy strategies are still lacking. It is essential and crucially important for vascular biologists to study fresh mechanisms and to seek out therapeutic targets of KD-induced vascular endothelium damage, which are all crucial for both the investigation and the clinical therapy of KD.

Several research studies have been conducted to investigate the origin of KD and its correlation with the diseased state in patients (14–16). It has already been well-recognized that they are accompanied by numerous cellular inflammatory responses in the peripheral blood, composed of the activation of white blood cells and the release of their contents into the blood circulation in KD pathogenesis. As was previously reported, endothelial damage often occurs in the early stage of a coronary artery aneurysm during the course of KD (6) and the biological functions of these contents in KD and the vascular injuries caused by KD are yet to be elucidated. Studies have shown positive correlations between the elevation of EMP levels and TNF-α expression in the peripheral blood of patients with KD, suggesting that EMPs are likely to play a crucial part in the development of vasculitis in patients with KD (13, 16).

It has been reported that IL-6 is a key inflammatory factor in regulating the inflammatory reaction in KD. IL-6 plays a vital role in innate immunity, adaptive immunity, acute and chronic inflammation, KD occurrence and development. IL-6 can quickly activate the downstream STAT3 signaling pathway through its receptor protein coupling gp130 (17, 18). In addition, both IL-1 and TNF-α could promote the secretion of IL-6 by upregulating NF-κB activity, thereby indirectly activating STAT3 (19, 20). IL-17 increases the levels of IL-6, thus indirectly activates the STAT3 signaling pathway, significantly inducing inflammation and promoting the occurrence of tumors (21–23). So, the IL-6/IL-6ST and STAT3 signaling pathway is of great significance for the regulation of the inflammatory response in KD.

In a previous study, a few researchers found that miRNAs in KD were statistically changed, especially miR-223-3p (13, 24). MiR-223-3p was seen to cause a significant decline in the production of IL-1β and IL-6, while the miR-223-3p inhibitor revealed the opposite role in regulating IL-1β and IL-6 production (25). STAT3 siRNA exhibits a similar effect in cells. Studies have shown that in TLR-triggered macrophages, miR-223-3p has the ability to regulate the STAT3 expression, which is closely related to the inflammatory reactions in macrophages during the microorganism infection. In addition, several researchers have also shown that in RAW 264.7 cells restraining STAT3 activity could hold back LPS-mediated IL-6 and IL-1β production as a priority, but not TNF-a (26). Data also proved that inhibiting STAT3 activity in gp130F/F mice, reduced the IL-6 expression level due to the response to LPS and then maintained the effect on STAT3 for promoting IL-6 gene transcription (27). All these studies indicate that the IL-6/STST3 signaling pathway plays a crucial role in the inflammatory responses.

KD is an important febrile illness causing multi-system vasculitis in childhood. Patients diagnosed with acute upper respiratory infection and herpangina, which mimic many of the clinical and laboratory findings in acute KD, were prospectively enrolled as febrile control subjects (14–16). In this research study, children with fever were selected as controls as well. We discovered that, miR-223-3p was highly expressed in the KD serum. The miR-223-3p expression level was high in acute stage KD and it was seen to decrease in coronary lesions of KD. IL-6 was confirmed as a target gene of miR-223-3p, and the miR-223-3p activator was thought to upregulate the expression of IL-6 and downregulate the expression of IL-6ST in KD mice. MiR-223-3p was observed to prohibit the expression of IL-6ST in an TNF-α induced inflammatory environment, and miR-223-3p was seen to regulate STAT3 and NF-κB p65 expression in the presence of TNF-α. This study yields an attractive molecular mechanism that indicates miR-223-3p's participation in KD pathogenesis through the adjustment and control of the IL6ST and STAT3 signaling pathways. Our research provides a reliable target gene for the clinical diagnosis and therapeutic treatment of Kawasaki disease.

## Data Availability

The raw data supporting the conclusions of this manuscript will be made available by the authors, without undue reservation, to any qualified researcher.

## Ethics Statement

This study was carried out in accordance with the recommendations of Medical ethics committee of Children's hospital of Soochow university and written informed consent was obtained from all subjects. All subjects gave written informed consent in accordance with the Declaration of Helsinki. The protocol was approved by the Medical ethics committee of Children's hospital of Soochow university.

The animal experiment study was also carried out in accordance with the recommendations of Medical ethics committee of Children's hospital of Soochow university. The related protocol were approved by medical ethics committee of Children's hospital of Soochow university too.

## Author Contributions

XW and YD contributed equally as co-first authors. XW contributed to designing the experiment and writing the manuscript. YD contributed to the design of the experiment. YC analyzed experimental results. QX established the animal model. GQ constructed the cell model. WQ performed statistic and analysis of data. LC performed bioinformatic analysis. WZ and MH collected and sorted out clinical case and experimental data. HL contributed to guiding, reviewing, inspecting of the experiments, and providing financial support.

### Conflict of Interest Statement

The authors declare that the research was conducted in the absence of any commercial or financial relationships that could be construed as a potential conflict of interest.
