# Corrigendum: MiR-223-3p Alleviates Vascular Endothelial Injury by Targeting IL6ST in Kawasaki Disease

**DOI:** 10.3389/fped.2019.00449

**Published:** 2019-11-05

**Authors:** Xiang Wang, Yue yue Ding, Ye Chen, Qiu qin Xu, Guang hui Qian, Wei guo Qian, Lei Cao, Wan ping Zhou, Miao Hou, Hai tao Lv

**Affiliations:** ^1^Department of Cardiology, Children's Hospital of Soochow University, Suzhou, China; ^2^Department of Pediatrics, The Affiliated Huaian No.1 People's Hospital of Nanjing Medical University, Huaian, China; ^3^Pediatric Research Institute of Soochow University, Suzhou, China

**Keywords:** Kawasaki disease, MicroRNA-223-3p, IL6ST, vascular endothelial damage, STAT3

In the original article, we neglected to include the funder “National Natural Science Foundation of China, No. 81970436” to “Yue yue Ding.”

A correction has been made to the **Funding** Statement:

“The article was financially supported by the National Natural Science Foundation of China (Nos. 81570455, 8187021691, 31600695, and 81970436), the Natural Science Foundation of Young (Nos. 81600391, 81800437, 81570439, 81400222), and the Jiangsu Province Science Foundation (BE2017660), the Talent Foundation of Jiangsu Province (ZDRCA2016049 and No. WSN-070), the Jiangsu Provincial Medical Young Talents (QNRC2016764, QNRC2016756) and the Applied Foundational Research of Medical and Health Care of Suzhou City (SYS201633, SYS201642).”

In addition, “Yue yue Ding” was not included as a co-first author in the published article. The corrected **Author Contributions** Statement appears below.

“XW and YD contributed equally as co-first authors. XW contributed to designing the experiment and writing the manuscript. YD contributed to the design of the experiment. YC analyzed experimental results. QX established the animal model. GQ constructed the cell model. WQ performed statistic and analysis of data. LC performed bioinformatic analysis. WZ and MH collected and sorted out clinical case and experimental data. HL contributed to guiding, reviewing, inspecting of the experiments, and providing financial support.”

The published article has also been updated to include the statement: “^†^These authors have contributed equally to this work.”

The authors apologize for these errors and state that they do not change the scientific conclusions of the article in any way. The original article has been updated.

