# Supplementary material for: MiR-223-3p Alleviates Vascular Endothelial Injury by Targeting IL6ST in Kawasaki Disease
Source: Front Pediatr. 2019 Jul 24;7:288. doi: 10.3389/fped.2019.00288 (PMC6667785; doi:10.3389/fped.2019.00288)
Supplement: Supplementary file 1 [file Data_Sheet_1.pdf]

Supplementary Materials

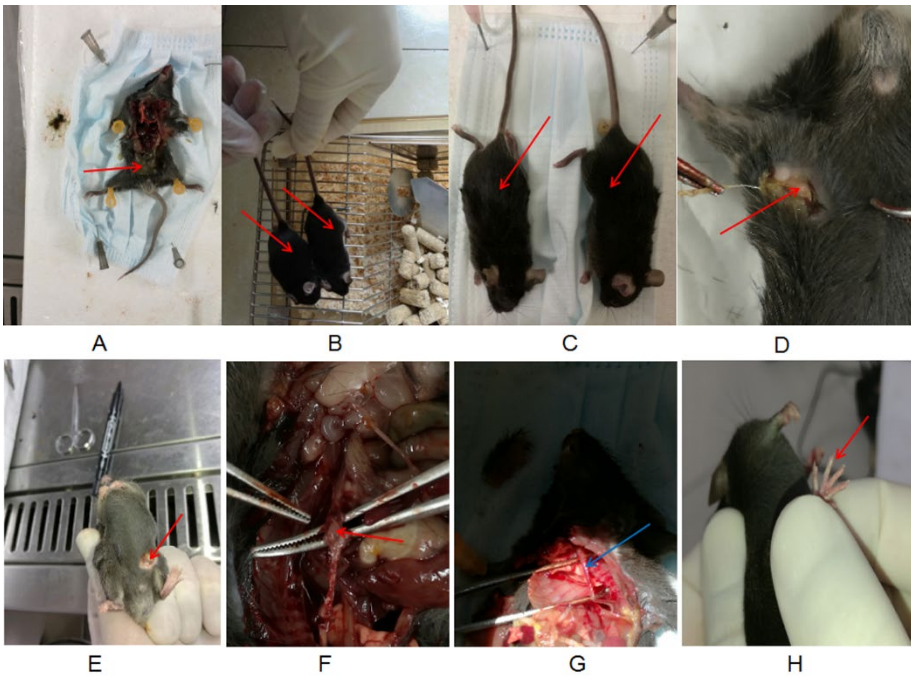

Supplementary Figure 1. The related pictures of KD mice model induced by intraperitoneal injection of CAWS.(A-H).

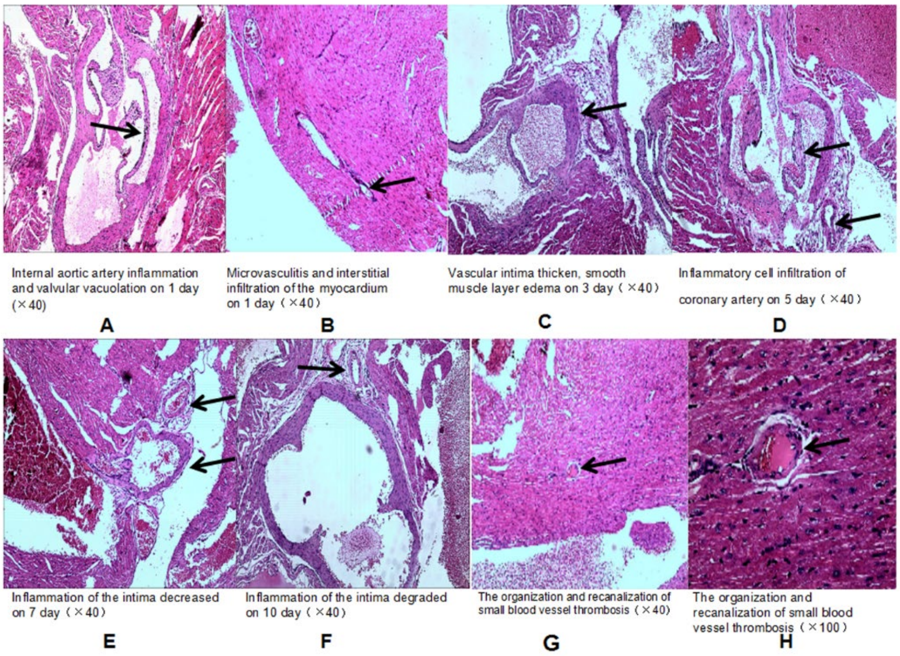

Supplementary Figure 2. Pictures related to HE staining of coronary artery specimens in KD mice induced by intraperitoneal injection of CAWS.(A-H).
